# Supplementary material for: The Neurotrophin Receptor TrkC as a Novel Molecular Target of the Antineuroblastoma Action of Valproic Acid
Source: Int J Mol Sci. 2021 Jul 21;22(15):7790. doi: 10.3390/ijms22157790 (PMC8346142; doi:10.3390/ijms22157790)
Supplement: Supplementary file 1 [file ijms-22-07790-s001.zip › ijms-1274190-supplementary.pdf]

International Journal of *Molecular Sciences*

Article

The neurotrophin receptor TrkC as a novel molecular target of the antineuroblastoma action of valproic acid

Simona Dedoni [1], Luisa Marras [2], Maria C. Olianas [1], Angela Ingianni [2] and Pierluigi Onali [1,*]

[1] Laboratory of Cellular and Molecular Pharmacology, Section of Neurosciences; dedoni@unica.it (S.D.); mariolina.olianas@gmail.com (M.C.O.)
[2] Section of Microbiology, Department of Biomedical Sciences, University of Cagliari, 09042 Monserrato, Italy; ; luisamarras@yahoo.it (L.M.); ingianni@unica.it (A.I.)
* Correspondence: onali@unica.it

Supplementary Figures

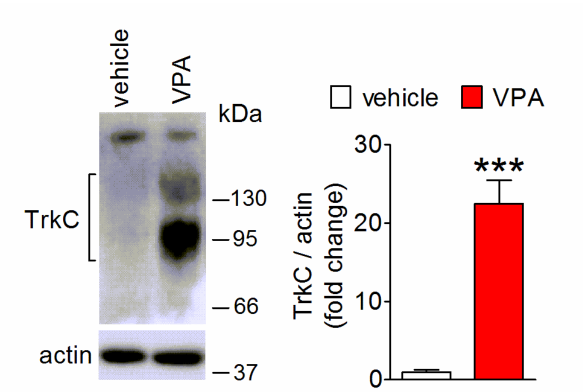

Figure S1. Sustained induction of TrkC by VPA exposure. SH-SY5Y cells were incubated in growth medium containing 10 % FCS with either vehicle or 0.6 mM VPA for 72 h and then analyzed for TrkC expression by Western blot. Values are the mean ± SD of four experiments. *** $p <$ 0.001 vs control (vehicle-treated cells) by Student's t test.

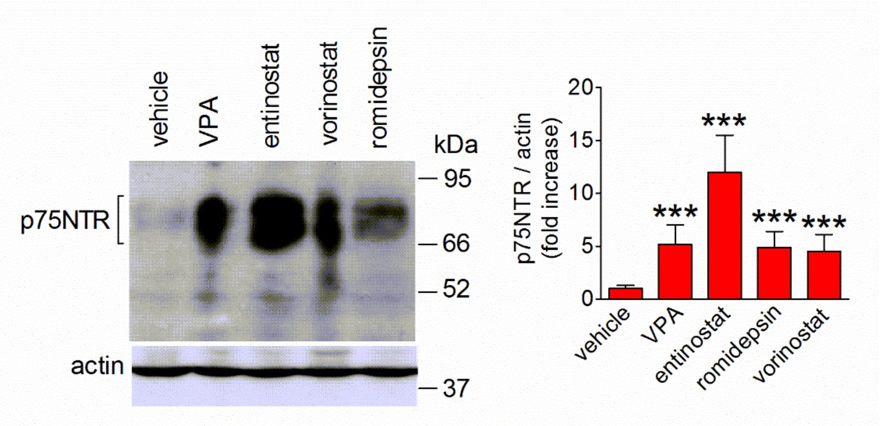

Figure S2. Exposure to HDAC inhibitors upregulates p75NTR expression in IMR 32 neuroblastoma cells. Cells were incubated for 24 h with either vehicle, 1 mM VPA, 1 µM entinostat, 20 nM romidepsin or 200 nM vorinostat. Cell lysates were analyzed for p75NTR and actin levels by Western blot. Values are the mean ± SD of four independent experiments. *** $p < 0.001$ vs control (vehicle) by ANOVA followed by Tukey's test.
